# Prevalence, clinical features and emergency department blood pressure management of hypertensive emergencies and uncontrolled hypertension: a retrospective multi-centre study

**DOI:** 10.1038/s41440-026-02788-1

**Published:** 2026-09-02

**Authors:** Alessandro Maloberti, Giulia Capitoli, Tommaso Valobra, Paola Rebora, Nicolas Mavreas, Silvia Vergani, Valentina Giani, Chiara Tognola, Nicolò Capsoni, Michele Galasso, Lorenzo De Censi, Cecilia Ferretti, Andrea Sultana, Gabriele Peraro, Martina Morelli, Annalisa Giacalone, Ilaria Garofani, Filippo Galbiati, Michele Bombelli, Cristina Giannattasio

**Affiliations:** 1 School of Medicine and Surgery, Milan-Bicocca University, Milan, Italy; 2 Cardiology 4, ASST Niguarda Ca Granda Hospital, Milan, Italy; 3 Bicocca Bioinformatics Biostatistics and Bioimaging (B4) Centre, School of Medicine and Surgery, University of Milano-Bicocca, Monza, Italy; 4 Biostatistics and Clinical Epidemiology, Fondazione IRCCS San Gerardo dei Tintori, Monza, Italy; 5 Pio XI Hospital of Desio, Internal Medicine Department, ASST Brianza, Desio, Italy; 6 Emergency Department, ASST Niguarda Ca Granda Hospital, Milan, Italy

**Keywords:** Hypertensive emergency, Uncontrolled hypertension, Morning hypertension, Implemental hypertension, Digital hypertension

## Abstract

Very heterogeneous data have been published concerning prevalence and clinical management of Hypertensive Emergencies (HE) and Uncontrolled Hypertension (UH). The aim of our study was to characterize HE and UH patients evaluating: (i) Blood Pressure (BP) management in Emergency Department (ED), the pharmacological treatment and its adherence to guidelines recommendation; (ii) variables associated with the presence of organ damage; and (iii), as an exploratory analysis, the impact of BP on in-hospital mortality. We performed a multicentre (Niguarda Hospital - Milan and Pio XI Hospital – Desio) retrospective study evaluating patients admitted to ED with BP values ≥ 180/120 mmHg during 2017 and 2019. 1838 patients were admitted to the ED with a BP ≥ 180/120 mmHg (0.95% of total admissions) of whom 37.7% were HE and the remaining (62.3%) were UH. The reduction in Systolic and Diastolic BP (SBP/DBP respectively) was similar in HE (40.6/16.9 ± 26.3/17.7 mmHg) and UH (39.3/15.5 ± 22.4/16.0 mmHg). The most used drugs were intravenous furosemide, nitroglycerin, clonidine and labetalol for HE. Age, smoking, heart failure, previous acute coronary syndrome, chronic kidney disease and diabetes mellitus present a positive association HEs while female sex and chronic anti-hypertensive therapies with 2 or more drugs results as protective variables. Finally, regarding the exploratory analysis, BP at ED admission and its management didn’t present significant association with in-hospital mortality. In conclusions, our study demonstrated that adherence to guidelines in the management of HE/UH could be further improved.

**Figure:**
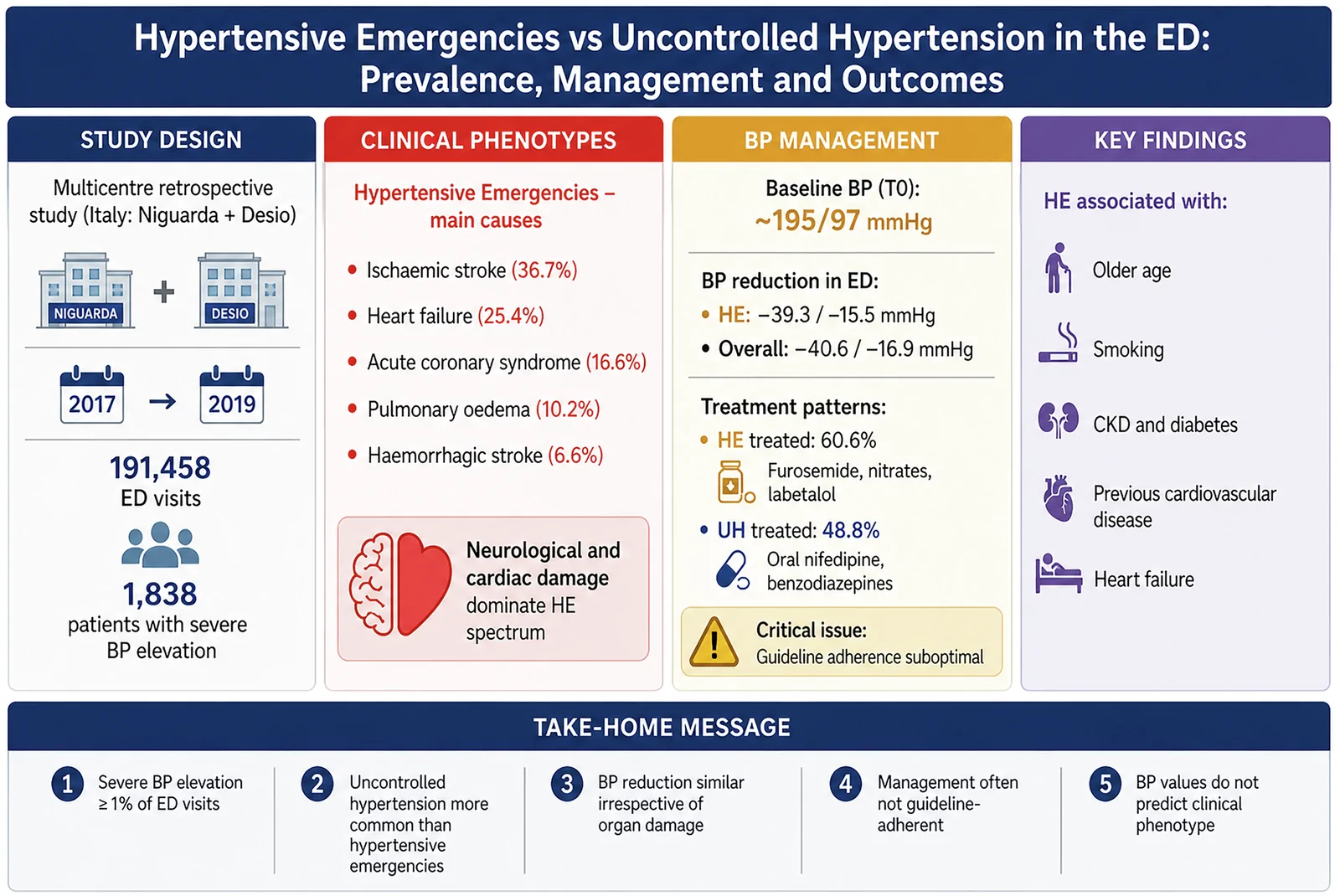

## Introduction

Hypertension (HT) is the leading modifiable cause of Cardiovascular (CV) morbidity and mortality [1]. Its diagnosis and treatment are clearly codified in international guidelines, while few evidence-based recommendations exist regarding the management of severely and acutely raised Blood Pressure (BP) values [2]. If HT Mediated Organ Damage (HMOD) is associated they are called Hypertensive Emergency (HE) with the risk of significant prognostic consequences if left untreated [3–5]. If HMOD is not present, irrespective of the severely elevated BP values, the patient is not considered at risk of immediate harm. This condition has been previously called hypertensive urgencies while latest guidelines proposed to change it to Uncontrolled HT (UH) because of the lack of prognostic consequences [3–5]. While European guidelines set a threshold of ≥180/110 mmHg for HE/HU definitions [3, 4], the American one defined them as a BP value ≥ 180/120 mmHg [5].

HMOD can be heterogeneous: CV (acute coronary syndrome, unstable angina pectoris, aortic dissection, pulmonary oedema, heart failure), neurological (ischaemic and hemorrhagic stroke), renal (acute kidney injury), eclampsia/pre-eclampsia and hypertensive encephalopathy [6]. They all require rapid diagnosis in order to set up timely intravenous treatment aimed at stop the progression of HMOD and reduce short and long-term morbidity and mortality [3–5]. In contrast UHs require slower reductions of BP values with oral medication and antihypertensive home treatment intensification [3–5].

We performed a multi-center study to investigate the characteristics of patients presenting to the Emergency Department (ED) with HE or UH. We evaluated: (i) BP management in ED, the pharmacological treatment and its adherence to guidelines recommendation; (ii) variables associated with the presence of organ damage; and (iii), as an exploratory analysis in hospitalized patients, the association of BP and in-hospital mortality. Since most of the epidemiological HE/UH studies use the ≥180/120 mmHg threshold we used this one in order to increase reproducibility.

## Methods

### Study population

We performed a multicentre retrospective study in two north Italian big hospital: ASST Grande Ospedale Metropolitano Niguarda (Milan, 90,000 ED access/year) and Papa Pio XI Hospital (Desio, 65,000 access/year). We collected data regarding all adult patients (age ≥18 years) admitted to the ED during the years 2017 and 2019 and discharged with at least one of the following diagnoses (and the relative International Classification of Disease – ICD- coding): heart failure (ICD 4280), pulmonary oedema (ICD 4281), myocardial infarction (with ST segment elevation – ICD 41091 – and without – ICD 41071), unstable angina pectoris (ICD 4111), stable angina pectoris (ICD 4139), cerebral ischaemia (ICD 43401), cerebral hemorrhage (ICD 430), subarachnoid hemorrhage (ICD 434), specified intracranial hemorrhage (ICD 4372), transient ischaemic attack (ICD 4359), hypertensive encephalopathy (ICD 4372), aortic dissection (ICD 44100), thoracic aortic aneurysm (ICD 4412), rupture of abdominal aortic aneurysm (ICD 4416), headache (ICD 7840), epistaxis (ICD 7847), anxiety (ICD 30000), vertigo (ICD 3869) and HT (ICD 4019).

Among selected subjects, all patients with a SBP ≥ 180 mmHg and/or a DBP ≥ 120 mmHg at their first BP measurement in the ED were included in the analysis. ED reports, inpatients and discharge document (in case of hospitalization) were reviewed for data collection and to correctly assign patients to the HE or UH group depending on the presence or absence of HMOD.

Pregnant women were excluded from the enrollment, both due to the peculiarity of their condition and their management in a specialist ED.

The following data were collected for each patient: age, gender, active smoking, clinical history (with particular regards to hypertension, cardiological history and CV risk factors as well as antihypertensive therapies), symptoms at presentation to ED, BP values and its management. In particular, BP was measured in a sitting position (whenever possible depending on patients’ clinical condition) and with a cuff size adequate to the arm circumference. The device available in our ED were Philips Intellivue monitor system (Amsterdan Netherlands) and three measurements were done with the mean values appointed on the ED reports.

BP values were systematically measured at ED presentation (T0) while subsequent values (1 to 2 other measurements) were not systematically assessed but is determined by physicians’ decision and varies depending on clinical presentation, diagnostic work-up and therapies administered. 383 patients had a missing in further BP values with only ED presentation one available (clinical characteristics shown in supplementary table 1). Of the remaining subjects, 1281 had 2 (T0 and another one) ED BP evaluations and 174 had 3 (T0 and other two) ED BP evaluations.

For the principal analysis regarding BP management, we considered T0 values and the lower values, among the available one, during the ED stay (T1). Since some patients had more than two ED BP values, as a sensitivity analysis, also the difference between T0 BP values and the higher further ED BP values measured was evaluated. Also time from presentation BP and the one considered for the analysis were calculated. Finally, we recorded if patients were treated with antihypertensive medication and eventually the specific drug used.

Laboratory tests in ED were performed at discretion of the physician. For that reason and for the clinical heterogeneity of HE and UH, data on biochemical variables are mostly missing and were not systematically collected.

Finally, all cause in-hospital mortality was evaluated in hospitalized patients. Study protocol was approved by ethics review committee of the institution involved.

### Diagnostic workup and operational definition

Patients’ diagnostic workup was defined by the ED physicians and guided by presentation symptoms and signs. It involved a comprehensive imaging and laboratory examinations including: 12-lead electrocardiogram, transthoracic echocardiography, chest radiography, biochemical urine dipstick analysis, funduscopic examination, head and contrast-enhanced chest computed tomography.

When HMOD were completely excluded, patients will then fall into the UH group. On the opposite, when HMOD was confirmed (based on the following operation definitions), it needs to be associated to a BP values ≥ 180/120 mmHg to identify the relative HE.

Acute coronary syndrome was diagnosed according to the Fourth Universal Definition of Myocardial Infarction, as the presence of acute myocardial injury with clinical evidence of acute myocardial ischaemia [7]. This was characterized by the detection of a rise or fall of Troponin C levels, with at least one value above the 99th percentile upper reference limit (> 14 ng/L) and at least one of the following criteria: (i) symptoms of myocardial ischaemia; (ii) new ischaemic Electrocardiographic (ECG) changes; (iii) development of pathological Q waves; (iv) imaging evidence of new loss of viable myocardium or new regional wall motion abnormalities in a pattern consistent with an ischaemic aetiology; and (v) identification of a coronary thrombus via angiography.

Acute pulmonary oedema was diagnosed as the presence of dyspnea with orthopnoea, respiratory failure (hypoxemia-hypercapnia), tachypnoea (>25 breaths/min), and increased work of breathing [8].

Heart failure was diagnosed as the presence of specific symptoms (dyspnea) and/or signs (pulmonary congestion, peripheral oedema) associated with elevated natriuretic peptides values and without the acute pulmonary oedema criteria [8].

Aortic dissection diagnosis was defined on radiological findings from a thoracic and/or abdomical computer tomography scan [9].

Ischaemic strokes were defined as the presence of neurological general or focal sign and symptoms associated with the presence of recent ischemic area at head computer tomography or early sign of brain arterial thrombus at contrast-enhanced head computer tomography. When these imaging evidences were not present but symptoms are focal and lasting patients need to be admitted to neurological department with a diagnosis of ischaemic stroke that coiuld be further evaluated during the admission [10].

When neurological symptoms were associated with the radiological evidence of brain hemorrhages a final diagnosis of hemorrhagic stroke was done.

Hypertensive encephalopathy consists of diffuse (not focal) cerebral symptoms determined by sustained BP rises that reverse when it is lowered and are not due to cerebral infarction or hemorrhage nor other known causes (infective, hepatic, glucose-related) [11]. This diagnosis needs a neurological confirmation after an inpatient’s diagnostic and therapeutic work-up.

Similarly, hypertension mediated acute renal failure needs to be confirmed by a prompt improvement with BP values reduction and exclusion of other possible causes by a nephrologist.

### Statistical analysis

Data were described by means and standard deviation or frequencies and percentages according to their nature and comparison between values in HE and UH group were done by Welch two sample t-test or Pearson’s Chi-squared test. Fisher’s exact test was used to compare the pharmacological treatment of patients with HE and UH and in-hospital mortality. Multivariable logistic regression model was used to evaluate the variables associated to the presence of HMOD including pre-specified baseline factors. The results were presented as Odds Ratio (OR) with 95% Confidence Interval (CI).

## Results

### Population characteristics

**Fig. 1:**
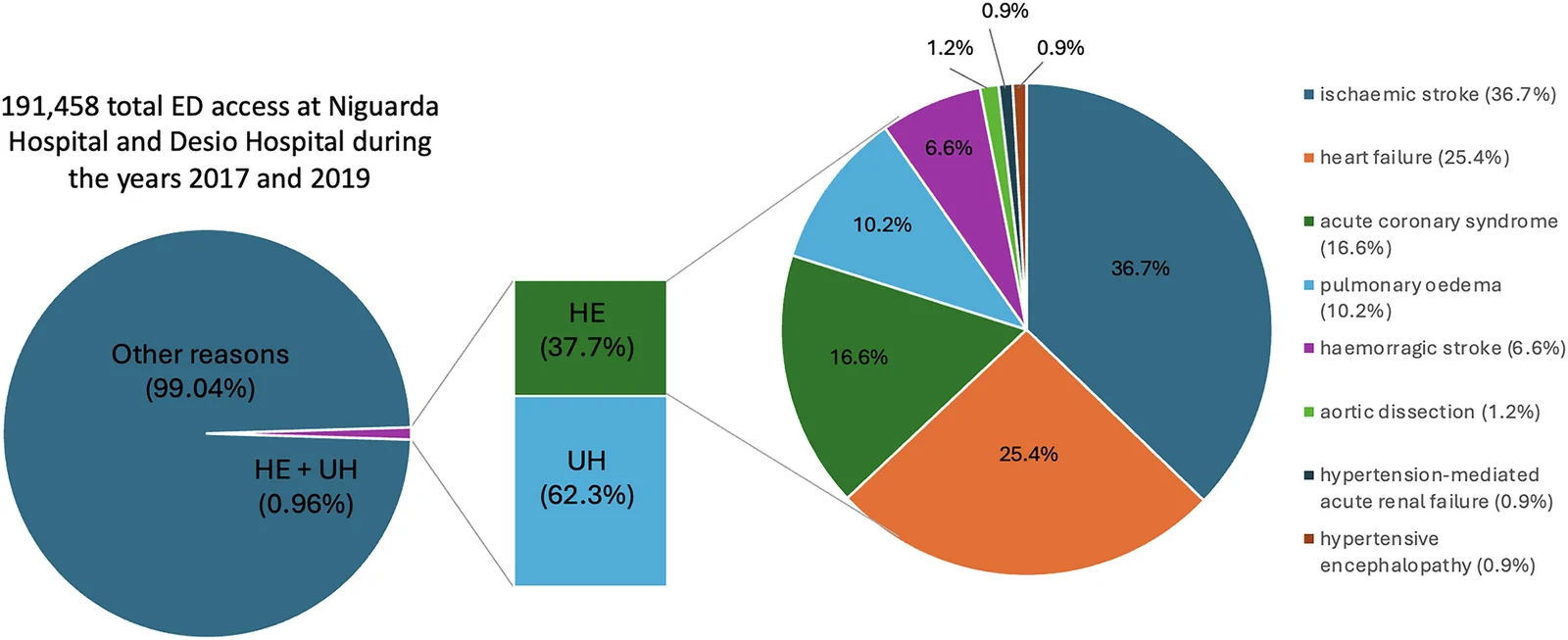
Prevalence of hypertensive emergencies and uncontrolled hypertension (and their sub-type)

As showed in Fig. 1, 1838 admission for HE/UH were found, representing the 0.96% of ED total accesses of which 1145 (62.3%) were UH and 693 (37.7%) were HE. Prevalence of HE sub-type were (Fig. 1): ischaemic stroke (36.7%), heart failure (25.4%), acute coronary syndrome (16.6%), pulmonary oedema (10.2%), hemorrhagic stroke (6.6%), hypertension-mediated acute renal failure (0.9%), hypertensive encephalopathy (0.9%) and aortic dissection (1.2%). Few patients had two HE sub-type at the same moment: two patients had both acute coronary syndrome and pulmonary oedema, three patients had heart failure and acute kidney injury while six patients had an ischemic stroke together with cerebral hemorrhage.

Table 1 showed the patient’s characteristics overall and by the presence/absence of HMOD (HE and UH). Mean age of the whole population was 70.5 ± 14.4 years, with 53.6% females. 73.3% of the population had a diagnosis of HT with 63.6% that were on anti-hypertensive medications (40.6% of them were on two or more drugs). Regarding other CV risk factors, 20.8% had diabetes and 11.2% and 11.0% had chronic kidney disease and smoking respectively. 88.4% of the patients were symptomatic being the most frequent one neurological deficit (22.9%).

**Table 1:** Clinical characteristics of the total sample and in patients classified as Hypertensive Emergencies and Uncontrolled Hypertension

Variable	Overall	Hypertensive emergencies	Uncontrolled hypertension	p-value
**Number**	1838	693	1145	
**Cardiovascular risk factors**				
**Age (years)**	70.5 ± 14.4	74.3 ± 12.5	68.2 ± 15.0	<0.001
**Females**	985 (53.6%)	331 (47.8%)	654 (57.1%)	<0.001
**Smoke**	203 (11.0%)	113 (16.3%)	90 (7.9%)	<0.001
**Diabetes**	382 (20.8%)	206 (29.7%)	176 (15.4%)	<0.001
**Hypertension**	1347 (73.3%)	527 (76.0%)	820 (71.6%)	0.037
**Chronic antihypertensive treatment**	1169 (63.6%)	478 (69.0%)	691 (60.3%)	<0.001
**Two or more antihypertensive drugs**	747 (40.6%)	313 (45.2%)	434 (37.9%)	0.002
**Cardiovascular and general comorbidities**				
**Chronic kidney disease**	205 (11.2%)	140 (20.2%)	65 (5.7%)	<0.001
**Peripheral artery disease**	290 (15.8%)	145 (20.9%)	145 (12.7%)	<0.001
**Previous acute coronary syndrome**	368 (20.0%)	203 (29.3%)	165 (14.4%)	<0.001
**Previous heart failure**	117 (6.4%)	97 (14.0%)	20 (1.7%)	<0.001
**Chronic obstructive pulmonary disease**	110 (6.0%)	75 (10.8%)	35 (3.1%)	<0.001
**Asthma**	32 (1.7%)	14 (2.0%)	18 (1.6%)	0.477
**Previous stroke**	147 (8.0%)	76 (11.0%)	71 (6.2%)	<0.001
**Cancer**	206 (11.2%)	94 (13.6%)	112 (9.8%)	0.013
**Emergency department symptoms**				
**Symptoms at presentation**	1625 (88.4%)	676 (97.5%)	949 (82.9%)	<0.001
**Chest pain**	328 (17.8%)	180 (26.0%)	148 (12.9%)	<0.001
**Dyspnea**	327 (17.8%)	253 (36.5%)	74 (6.5%)	<0.001
**Syncope**	28 (1.5%)	8 (1.2%)	20 (1.7%)	0.315
**Neurological Deficit**	420 (22.9%)	289 (41.7%)	131 (11.4%)	<0.001
**Palpitation**	73 (4.0%)	20 (2.9%)	53 (4.6%)	0.064
**Headaches**	367 (20.0%)	48 (6.9%)	319 (27.9%)	<0.001
**Vertigo**	260 (14.1%)	37 (5.3%)	223 (19.5%)	<0.001
**Vomit**	118 (6.4%)	37 (5.3%)	81 (7.1%)	0.141
**Epistaxis**	118 (6.4%)	1 (0.1%)	117 (10.2%)	<0.001
**Abdominal pain**	25 (1.4%)	3 (0.4%)	22 (1.9%)	0.008

Data are shown as mean ± standard deviation or number and percentages for continuous and categorical one, respectively

Welch Two Sample t-test or Pearson’s Chi-squared were used for comparison

611 patients (33.2%) were hospitalized with a mean length of stay of 10.8 ± 8.5 days while 28 patients (4.6%) died during ward stay.

### Hypertensive emergencies vs. uncontrolled hypertension patients

As shown in Table 1, HE patients were older (74.3 ± 12.5 vs. 68.2 ± 15.0 years, *p* < 0.001), more frequently males (52.2 vs. 42.9%, *p* < 0.001) and smokers (16.3 vs. 7.9% *p* < 0.001) and with more comorbidities such as chronic kidney disease, diabetes mellitus, peripheral arterial disease, previous CV events (acute coronary syndrome, stroke and heart failure), cancer and chronic obstructive pulmonary disease. Furthermore, they had more frequently an HT diagnosis (76.0 vs. 71.6%, *p* = 0.037) and took more anti-hypertensive drugs (69.0 vs. 60.3%, *p* < 0.001), particularly, 2 or more of them (45.2 vs. 37.9% *p* = 0.002).

Patients with HE were more often symptomatic at ED presentation (97.5 vs. 82.9%, *p* < 0.001). The most frequent presenting symptom in HE patients was neurologic deficit (41.7%), followed by dyspnea (36.5%) and chest pain (26.0%); while in patients with UH the most frequent symptom was headache (27.9%), followed by vertigo (19.5%), neurological deficit (11.4%) and epistaxis (10.2%).

HE patients were frequently hospitalized while UH patients were not (80.8 vs. 4.5%, *p* < 0.001). BP data of hospitalized vs. not-hospitalized patients, hospitalized HE vs. UH and survivor’s vs. in-hospital death were reported in Supplementary Table 2. Hospitalized patients (*n* = 611) shown similar baseline BP values and ED BP decrease when compared to not-hospitalized subjects. Among hospitalized patients there was no significant difference in terms of length of stay (10.7 ± 7.4 vs. 11.6 ± 16.0 days, *p* = 0.702) and in-hospital mortality (4.8 vs. 2.0%, *p* = 0.501) between HE and UH. The same also applies to BP values (196.1/96.7 ± 16.3/16.3 vs. 196.7/95.2 ± 18.3/19.1 mmHg for HE and UH respectively, *p* = 0.814 and 0.600 for SBP and DBP respectively) and their decrease during ED period (40.6/17.9 ± 27.1/17.9 vs. 40.2/16.0 ± 25.7/19.1 for HE and UH respectively, *p* = 0.924 and 0.762 for SBP and DBP respectively) that shown superimposable values. Finally, in the same group, BP values at T0 (195.9/96.6 ± 16.2/16.4 mmHg in patients that have been discharge alive and 200.9/95.7 ± 20.6/19.4 mmHg in patients who died in-hospital, *p* = 0.217 and 0.811 for SBP and DBP respectively) and their variation during ED stay (40.9/17.1 ± 26.8/17.9 vs. 33.7/13.3 ± 33.1/23.2 mmHg, *p* = 0.391 and 0.513 for SBP and DBP respectively) did not show any significant difference regarding to in-hospital mortality.

### Blood pressure management in the emergency department

As shown in Table 2, in the whole population, BP at ED presentation (T0) was 194.5/97.0 ± 15.1/16.4 mmHg and 53.3% of the patients were treated in ED. The most frequent pharmacological treatments were Furosemide (14.9%) and Clonidine (12.1%, Table 3). For a total of 383 patients (140 HE and 243 UH) no other BP values following the first one at ED admission were reported, thus analysis on BP management were done on 1455 patients. In these patients, the BP decrease resulted 39.8/16.1 ± 23.9/16.6 mmHg with higher values in treated patients (44.8/19.2 ± 24.5/17.4 mmHg vs. 32.3/11.3 ± 20.9/14.1).

**Table 2:** Blood pressure values at presentation (T0) and decrease at ED in in the total sample and in patients classified as Hypertensive Emergencies and Uncontrolled Hypertension

Variable	Overall	Hypertensive emergencies	Uncontrolled hypertension	p-value
**Number of patients**	1838	693	1145	
**SBP at presentation (mmHg)**	194.5 ± 15.1	195.4 ± 16.0	193.9 ± 14.5	0.050
**DBP at presentation (mmHg)**	97.0 ± 16.4	96.2 ± 16.2	97.5 ± 16.5	0.115
**Number of patients with a second BP measure**	1455	553	902	
**SBP decrease (mmHg)**	39.8 ± 23.9	40.6 ± 26.3	39.3 ± 22.4	0.312
**DBP decrease (mmHg)**	16.0 ± 16.6	16.9 ± 17.7	15.5 ± 15.9	0.140
**Time from the two considered BP evaluations (minutes)**	221.1 ± 183.7	226.3 ± 220.3	217.3 ± 151.4	0.505
**Patients treated in the emergency department**				
**Number of patients treated in ED**	979 (53.3%)	420 (60.6%)	559 (48.8%)	<0.001
**SBP at presentation (mmHg)**	198.5 ± 16.5	197.8 ± 17.4	199.1 ± 15.7	0.199
**DBP at presentation (mmHg)**	101.5 ± 17.2	99.1 ± 17.3	103.3 ± 17.0	<0.001
**Number of patients treated in ED with a second BP measure**	873	372	501	
**SBP decrease (mmHg)**	44.8 ± 24.5	44.2 ± 26.9	45.2 ± 22.6	0.564
**DBP decrease (mmHg)**	19.2 ± 17.4	19.5 ± 18.5	18.9 ± 16.6	0.644
**Time from the two considered SBP evaluations (minutes)**	221.0 ± 179.8	225.0 ± 212.1	218.0 ± 151.9	0.601
**Patients untreated in the emergency department**				
**Number of patients untreated in ED**	859 (46.7%)	273 (39.4%)	586 (51.2%)	-
**SBP at presentation (mmHg)**	189.8 ± 11.9	191.7 ± 12.9	188.9 ± 11.3	0.001
**DBP at presentation (mmHg)**	92.0 ± 13.8	91.9 ± 13.5	92.0 ± 14.0	0.982
**Number of patients untreated in ED with a second BP measure**	582	181	401	
**SBP decrease (mmHg)**	32.3 ± 20.9	33.2 ± 23.4	31.8 ± 19.7	0.486
**DBP decrease (mmHg)**	11.3 ± 14.1	11.5 ± 14.3	11.2 ± 14.0	0.859
**Time from the two considered SBP evaluations (minutes)**	226.7 ± 346.9	302.8 ± 548.2	175.9 ± 124.8	0.600

Data are shown as mean ± standard deviation or number and percentages for continuous and categorical one, respectively. Welch Two Sample t-test or Pearson’s Chi-squared were used for comparison. 383 patients had a missing in further BP values with only ED presentation one available

*ED* Emergency Department, *SBP* Systolic Blood Pressure, *DBP* Diastolic Blood Pressure

**Table 3:** Pharmacological treatment in the total sample and in patients classified as Hypertensive Emergencies and Uncontrolled Hypertension

Variable	Route of administration	Overall	Hypertensive emergencies	Uncontrolled hypertension	p-value
**Number of patients**	-	1838	693	1145	**-**
**Nitroglycerin**	Intravenous	102 (5.5%)	93 (13.4%)	9 (0.8%)	<0.001
**Nitroglycerin**	Sublingual	45 (2.4%)	35 (5.1%)	10 (0.9%)	<0.001
**Metoprolol**	Intravenous	19 (1.0%)	14 (2.0%)	5 (0.4%)	0.002
**Labetalol**	Intravenous	47 (2.6%)	41 (5.9%)	6 (0.5%)	<0.001
**Urapidil**	Intravenous	20 (1.1%)	18 (2.6%)	2 (0.2%)	<0.001
**Furosemide**	Intravenous	273 (14.9%)	233 (33.6%)	40 (3.5%)	<0.001
**Clonidine**	Intravenous	222 (12.1%)	48 (6.9%)	174 (15.2%)	<0.001
**Bisoprolol**	Oral	17 (0.9%)	14 (2.0%)	3 (0.3%)	<0.001
**Atenolol**	Oral	11 (0.6%)	5 (0.7%)	6 (0.5%)	0.756
**Captopril**	Oral	67 (3.6%)	9 (1.3%)	58 (5.1%)	<0.001
**Ramipril**	Oral	21 (1.1%)	12 (1.7%)	9 (0.8%)	0.072
**Enalapril**	Oral	25 (1.4%)	10 (1.4%)	15 (1.3%)	0.837
**Nifedipine short acting**	Oral	150 (8.2%)	17 (2.5%)	133 (11.6%)	<0.001
**Nifedipine long acting**	Oral	12 (0.7%)	2 (0.3%)	10 (0.9%)	0.230
**Amlodipine**	Oral	151 (8.2%)	38 (5.5%)	113 (9.9%)	<0.001
**Doxazosin**	Oral	12 (0.7%)	7 (1.0%)	5 (0.4%)	0.148
**Benzodiazepines**	Oral	147 (8.0%)	24 (3.5%)	123 (10.7%)	<0.001

Data are shown as number and percentages. Fisher’s exact test was used for comparison

Patients with further ED BP values missing (Supplementary Table 1) shown a lower prevalence of chronic hypertension (64.7 vs. 75.5%, *p* < 0.001) and antihypertensive treatment (56.3 vs. 65.4%, *p* < 0.001) as well as a lower prevalence of previous heart failure diagnosis (3.7 vs. 7.1%, *p* = 0.015). Furthermore, a difference in symptoms at ED presentation was found with lower prevalence of dyspnea (14.1 vs. 18.7%, *p* = 0.034), neurological deficit (14.1 vs. 18.7%, *p* = 0.034), headaches (13.6 vs. 21.6%, *p* < 0.001) and vertigo (9.1 vs. 15.4%, *p* = 0.002). Their BP values at ED presentation were lower (187.5/90.6 ± 10.6/13.0 vs. 196.3/98.7 ± 15.6/16.8 mmHg, *p* < 0001 for both systolic and diastolic BP) and, finally, they were less frequently treated in ED (28 vs. 60%, *p* < 0.001) with less use of intra-venous nitroglycerin, labetalol, furosemide, clonidine, captopril, short acting nifedipine and amlodipine.

**Fig. 2:**
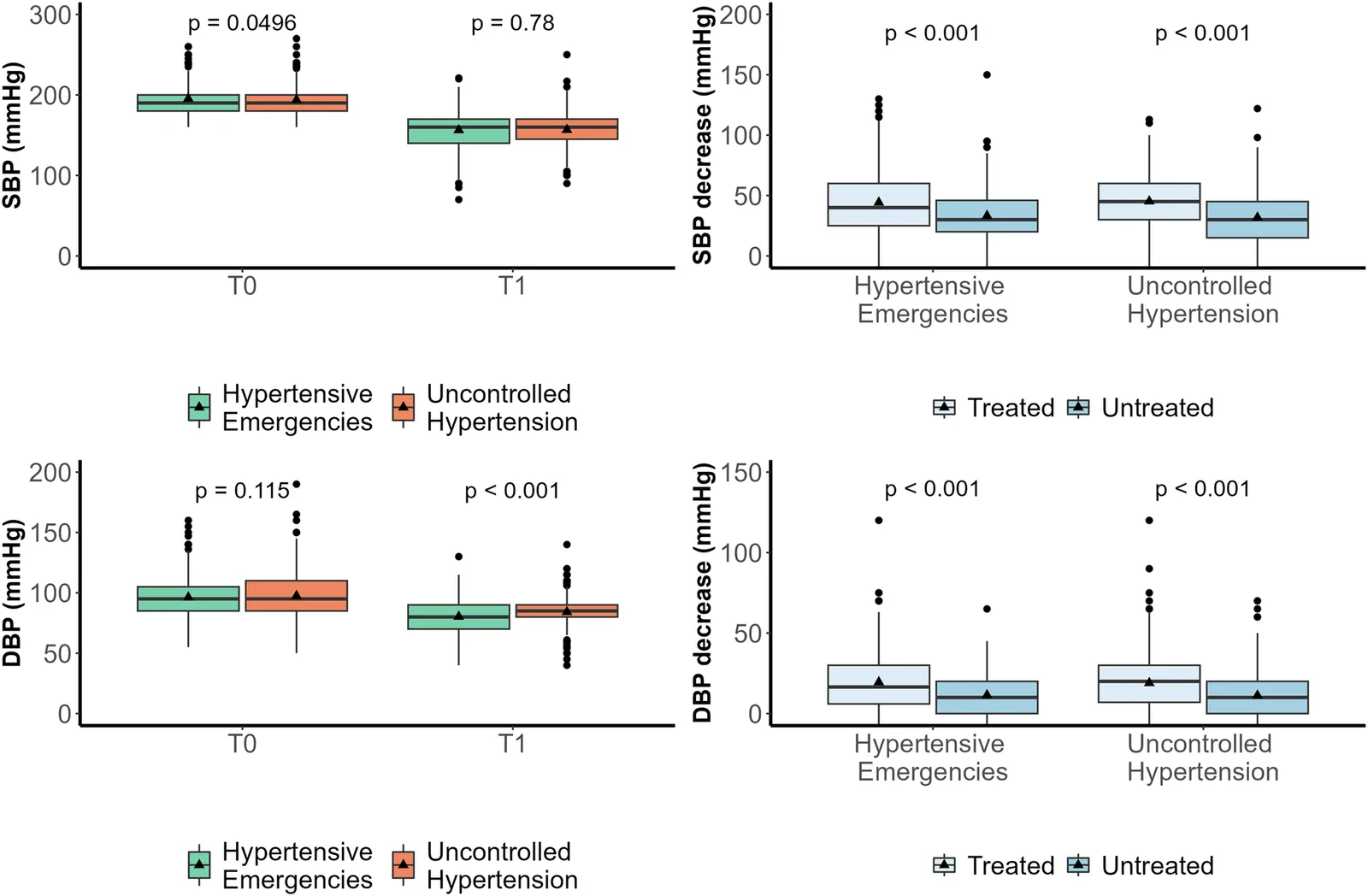
Systolic (panel **A**) and diastolic (panel **B**) blood pressure values at T0 and T1 and their relative delta based on treatment (panel **C** and **D**)

Analysing differences between HE and UH (Table 2), at T0 no difference was seen for SBP (195.4 ± 16.0 vs. 193.9 ± 14.5 mmHg, p = 0.05 – Fig. 2, panel **A**) and DBP (96.2 ± 16.2 vs. 97.5 ± 16.5 mmHg, *p* = 0.115 - Fig. 2, panel **B**). Similarly, also the mean total decrease during ED was similar in HE and UH (40.6/16.9 ± 26.3/17.7 vs. 39.3/15.5 ± 22.4/16.0, *p* = 0.312 and 0.140 for SBP and DBP respectively). Mean time from BP evaluation at T0 and T1 were 221.1 ± 183.7 mins without significant differences among HE and UH (226.3 ± 220.3 vs. 217.3 ± 151.4 min, *p* = 0.505).

When the higher second ED BP values available was considered instead of the lower one,

Results doesn’t change with a similar mean total decrease during ED stay in HE and UH (35.5/14.0 ± 25.8/17.0 vs. 36.9/14.4 ± 22.6/16.0, *p* = 0.298 and 0.686 for SBP and DBP respectively, Supplementary Fig. 1, panel A and B). For this analysis mean time between the two BP evaluation were 199.4 ± 173.0 mins and also in this case there were no significant differences among HE and UH (198.6 ± 203.5 vs. 200.0 ± 146.4 min, *p* = 0.911).

60.6% of the HE and 48.8% of the UH (*p* < 0.001) were treated with anti-hypertensive drugs. The most commonly used drugs in HE patients were intravenous furosemide (33.6%), nitroglycerin (13.4%), clonidine (6.9%), labetalol (5.9%) and urapidil (2.6%) while, among oral drugs, the most frequently used was amlodipine (5.5%). HU instead were mainly treated with oral drugs, including in particular short acting nifedipine (11.6%), benzodiazepines (10.7%), amlodipine (9.9%) and captopril (5.1%). Parenteral drugs were used rarely, with the exception of clonidine which was the most frequently used drug in HU (15.2%).

As expected, treated patients had a higher BP decrease when compared with untreated one (p < 0.001 for all comparison, Table 2 and Fig. 2 - panel C and D) while the BP decrease was similar in treated HE and UH as well as for untreated HE and UH (Table 2).

When the higher second ED BP values available was considered instead of the lower one, results were confirmed for UH and HE DBP (Supplementary Fig. 1, panel D) while differences were not still significant for HE SBP decrease (Supplementary Fig. 1, panel C).

A total of 273 HE patients (39.2%) were left untreated. Among them, 168 had an ischaemic stroke of whom only 8 were treated with thrombolysis. Five of them had BP values < 185/110 and so they were correctly left untreated. The other 3 had a second BP value during ED stay below the guideline’s threshold. Among patients not treated with thrombolysis, only 9 (5.6%) had a BP ≥ 220/120 mmHg and would therefore have required anti-hypertensive therapies according to guidelines recommendations. Although untreated, 6 of these patients had a second BP measurements during the ED stay with values below the guideline’s threshold. Consequently, the vast majority were appropriately left untreated fin accordance with guideline recommendations.

Furthermore, there were 21 untreated hemorrhagic strokes. All of them had an indication to anti-hypertensive treatment since baseline SBP values were ≥180 mmHg. However, only 2 of them, despite the absence of specific therapies, had a second BP values measured in ED still above 180 mmHg. Comparing treated and untreated subjects (Supplementary Table 3) no significant differences were seen nor regarding clinical characteristics nor for BP values.

6 acute kidney injury were left untreated. When compared to treated acute kidney injury (Supplementary Table 4) they were older (79.8 ± 10.1 vs. 74.3 ± 14.3 for untreated and treated patients respectively), had less frequently diabetes (16.6 vs. 42.8%) but more frequently chronic kidney disease (83.3 vs. 42.8%). Regarding BP, untreated patients had lower baseline values (185.0/86.7 ± 8.4/15.4 vs. 197.1/100.7 ± 22.3/21.9 mmHg for untreated and treated patients respectively) with a similar BP reduction despite the absence of a specific treatment (37.5/25.0 ± 3.5/7.1 vs. 37.0/12.0 ± 26.8/9.7 mmHg).

Only 2 acute pulmonary oedema were left untreated (supplementary table 5), age was not significantly different but these subjects doesn’t present any CV risk factors, comorbidities and any chronic anti-hypertensive therapies. They had slightly baseline lower BP values (180.0/92.5 ± 0.0/17.7 vs. 198.3/104.2 ± 18.3/18.0 for untreated and treated patients respectively) with significantly lower BP reduction (7.5/2.5 ± 17.7/10.6 vs. 55.6/29.3 ± 33.0/24.7 for untreated and treated patients respectively).

Among patients with a heart failure EH, 26 were not treated in ED (Supplementary Table 6). The only significant difference found between untreated and treated subjects is a lower value of DBP for the first group (88.0 ± 13.3 vs. 96.0 ± 17.2, *p* = 0.022).

Furthermore, 53 untreated patients had an acute coronary syndrome (Supplementary Table 7). They present a borderline significance for a reduced female (28.3 vs. 46.8%, *p* = 0.056) and chronic kidney disease (5.7 vs. 18.7%, *p* = 0.050) prevalence. Furthermore, untreated acute coronary syndrome had lower SBP (188.6 ± 8.6 vs. 194.8 ± 13.1 mmHg, *p* = 0.010) as well as lower SBP decrease (26.6 ± 17.5 vs. 45.2 ± 22.9 mmHg, *p* < 0.001).

Finally, all the aortic dissection therapies were not registered in ED and so they appear to be untreated. Nine untreated patients experienced more than one HE.

Mean time between BP measurements for treated and untreated patients were 221.0 ± 179.8 and 226.7 ± 346.9, respectively, without significant differences for HE and UH (Table 2).

### Multivariable analysis

At multivariable logistic regression (Table 4) we found that age (OR 1.03; 95% CI 1.02, 1.04; *p* < 0.001), smoking (OR 2.68; 95% CI 1.93, 3.74; *p* < 0.001), chronic kidney disease (OR 2.47; 95% CI 1.73, 3.56; *p* < 0.001), diabetes mellitus (OR 1.68; 95% CI 1.30, 2.18; *p* < 0.001), previous acute coronary syndrome (OR 1.63; 95% CI 1.25, 2.14; *p* < 0.001) and previous heart failure (OR 5.25; 95% CI 3.12, 9.19; *p* < 0.001) were positively associated with HMOD while female sex (OR 0.71; 95% CI 0.58, 0.89; *p* = 0.002), and chronic anti-hypertensive therapies with two or more drugs (OR 0.70; 95% CI 0.52, 0.94; *p* = 0.017) present a negative association.

**Table 4:** Factors associated with the presence of hypertension mediated organ damage according to multivariable logistic regression model

	Odds Ratio	95% Confidence Interval		p-value
		Lower limit	Upper limit	
**Age (per year)**	1.03	1.02	1.04	<0.001
**Female gender**	0.71	0.58	0.89	0.002
**Smoke**	2.68	1.93	3.74	<0.001
**Chronic Kidney Disease**	2.47	1.73	3.56	<0.001
**Diabetes**	1.68	1.30	2.18	<0.001
**Hypertension**	0.96	0.73	1.27	0.783
**Peripheral Artery Disease**	0.91	0.68	1.23	0.544
**Previous acute coronary syndrome**	1.63	1.25	2.14	<0.001
**Previous heart failure**	5.25	3.12	9.19	<0.001
**Chronic Obstructive Pulmonary Disease**	1.60	1.00	2.58	0.051
**Previous ischaemic stroke**	1.35	0.92	1.97	0.125
**Chronic antihypertensive therapy**	1.03	0.76	1.39	0.853
**Chronic antihypertensive therapy >=2**	0.70	0.52	0.94	0.017

Number of patients 1838, number of hypertensive emergencies 693

## Discussion

The first result of our study regards HE and UH epidemiological data. We found that approximately 1 in every 100 adult patients admitted to the ED had a severe BP elevation, that UH were more frequent than HE (62 vs. 38%) and that nearly 70% of the patients presenting with UH/HE had a prior diagnosis of HT. Although these findings are consistent with previous studies on the topic [12–14], some reports have described a lower prevalence (0.45–0.5%), likely due to the use of different threshold for defining severe BP elevation (i.e. ≥220/120 mmHg [15] or considering only a diastolic BP ≥ 120 mmHg [16]), as well as differences in patient selection methods (identifying patients on discharge diagnosis, as in our study, versus selecting patients according to BP values at admission, such as in the previously cited studies).

Secondly, from our study we can confirm variables predisposing to the occurrence of HE: advanced age, smoking and the presence of CV (diabetes mellitus, previous acute coronary syndrome, heart failure) and non-CV (chronic kidney disease and chronic obstructive pulmonary disease) co-morbidities. On the opposite female sex and chronic anti-hypertensive treatment were protective. Our findings are consistent with previously published studies also with regards to this latter aspect. In this context, some authors have suggested that discontinuation or poor adherence to antihypertensive therapy plays a significant role in the occurrence of HE/UH [14], although we were not able to further investigate this point.

Regarding sex, we found female sex as a protective factor for the occurrence of an HE. This finding contradicts some previously published works [15, 17, 18] although they are in line with other studies [16, 19]. The discrepancy could be due to differences in epidemiology, environmental, socio-cultural or genetic factors. Furthermore, it could be due to the fact that some studies using pregnancy as an exclusion criterion, which leads to underestimation of associated HE.

Regarding adherence to guidelines, some areas showed room for improvement, whereas others were largely consistent with recommendations. Specifically, we found that: (i) most patients with HE were hospitalized (80.8%) whereas the opposite was true for those with UH (4.5%); (ii) patients with stroke but without an indication for thrombolysis were generally not treated unless their BP values were markedly elevated (≥220/120 mmHg); (iii) intravenous therapies were predominantly used for HEs while oral agents were mainly administered for UHs; (iv) only half of UH patients received anti-hypertensive medication in the ED; and (v) the recommended 25% BP reduction was achieved in patients with HE.

Regarding the first point, UH hospitalizations were related to the coexistence of other conditions and not to the BP values themselves. On the contrary, HE patients that have not been hospitalized were cases of decompensated heart failure, in which fluid loss through diuretics, optimization of therapy and management of the trigger (e.g. supraventricular tachyarrhythmia) could be managed in the context of a short ED stay.

Regarding untreated patients, most of them were stroke (both ischaemic and hemorrhagic) that were not treated following guidelines recommendations. However, as also shown recently from our group [20], there is space for improvement also in this field despite guidelines are still very clear and evidences support their indications.

Furthermore, some acute coronary syndrome patients were not treated with anti-hypertensive therapies, despite guidelines. For this group of patients, guidelines [21] suggest an immediate reduction of BP to < 140/90 mmHg, however, it is possible that logistic issues underlie this finding, as acute coronary syndrome patients had to be taken to the catheterization laboratory. Some of them started an anti-hypertensive therapy in the ED but no subsequent BP values were available, while some other started a treatment during or after catheterization.

The same could also apply to hemorrhagic stroke in which surgical cases are very rapidly discharged from ED and take in charge by the anaesthetist and the neurological surgeon. So it could be possible that anti-hypertensive therapies administered were not registered on ED document leading to a misclassification bias.

Similarly, aortic dissections were all left untreated probably very quickly left to anesthetist and cardiac surgeon and data on they BP and its treatment were not recorded on the ED document.

Beside this point, when we compared untreated with treated subjects (within the specific EH) a common characteristic can be found for the first group: they present lower BP values. Furthermore, in some EH also an older age and prevalence of chronic kidney disease (for acute kidney injury) and a lower prevalence of CV risk factors (for acute pulmonary oedema) can be found in untreated patients.

When the higher second ED BP values available was considered instead of the lower one, the only significant variation regards to SBP decrease in HE which is not still significant when comparing treated and untreated patients. This is probably determined by the fact that the second measurement was too early to show a significant reduction and treatment intensification and further measurements are needed.

Two discrepancies with guidelines could be found in our study regarding anti-hypertensive treatment: labetalol (suggested as one of the first-line therapies) is used only in 5.9% of cases and short-acting nifedipine is too frequently used despite guidelines indicating to avoid it. This is determined by the fact that the degree of BP decrease, as well as the duration of its effects, is unpredictable and variable between patients. In some cases, the BP decrease observed could be disproportionate, resulting in hypotension and reflex tachycardia with development of adverse events (flushing, headache, palpitation and nausea) [22]. This is particularly of importance since it has been frequently used in UH, a subset of patients in which possible iatrogenic risks associated with rapid BP reduction surely overweight possible benefit raising ethical issue.

Other recent papers also found similar results of inconsistency between recommendations in the guidelines and real-life clinical practice [23, 24]. To overcome these points, educational initiatives should be undertaken to standardize treatment protocols and to improve medical adherence to guidelines recommendations.

Another point of our study which needs to be mentioned is the important BP reduction observed in untreated subjects. In fact, 50.4% of UH patients were not treated with anti-hypertensive drugs during their stay in the ED. In these patients, despite the absence of specific therapies, a BP reduction of 31.8/11.2 ± 19.7/14.0 mmHg was seen (and similar findings was found also in HE). These data raise the question whether anti-hypertensive therapies are really needed in UH while probably, in the absence of HMOD, an observational period of few hours could be the best management in such patients, especially if a significant emotional component is likely [5]. Regarding this, anxiety-related increase in BP determined by the acute setting and by the patient’s disease could determine classification in the UH group. Furthermore, BP measurements in ED are mostly inaccurate due to time limitations (only one measurement in most cases instead of the mean of 2 or 3 evaluations) and technical error could have a big influence on diagnosis. In fact, benzodiazepines were frequently used for the treatment of UH (10.7%) while its use in HE is more probably related to the need of pain relief or sedation for procedures. Finally, a recent study found that most of the patients that were discharged from an ED with an UH diagnosis had an adequate BP control at a 72-h visit despite no significant changes in chronic anti-hypertensive therapies [25].

These data, together with the very low probability of UH hospitalization, are in line with the guideline’s recommendation to avoid ED access for the vast majority of UH. In fact, asymptomatic patients don’t need HMOD exclusion and acute treatment and they can be appropriately managed in an outpatient setting. Avoiding unnecessary ED access, or provide a rapid discharge after ED triage with a subsequent outpatient’s quick visit could help reduce ED overcrowding and healthcare costs.

The last point we would like to discuss is our exploratory analysis regarding mortality, in which we found no significant differences nor for baseline BP values nor for their changes during ED stay. Our study is underpowered for this outcome (based on 28 deaths), while a further limitation could be that HE is a very heterogeneous group in which diseases with a high mortality rate (i.e. aortic dissection and hemorrhagic stroke) are grouped together with others that mostly have an excellent survival probability. So, our results should not be overinterpreted and future larger studies focused on single diseases are needed in order to define if appropriate BP management in ED could carry prognostic information. It is also possible that BP values don’t influence short-term in-hospital prognosis because the disease itself leading to hospitalization could be the main determinant of mortality and so longer follow-up are needed in future studies.

In fact, a recent study conducted in northern Italy showed that patients suffering a HE had a significantly higher 1-year risk of CV events when compared to UH (27.2 vs. 7.2/100 patient-years, respectively) [19]. In this study DBP (but not SBP) at ED access was found as one of the determinants of 1-year CV mortality.

Our study had several limitations, the most important one being its retrospective and cross-sectional design that didn’t allow us to calculate the incidence of the phenomenon, but only its prevalence. Also, in regard to this, only in-hospital longitudinal data were available while the absence of longitudinal long-term information prevented us from analysing the impact of acute BP management on subsequent outcome and recurrence. Secondly, subjects were primarily selected on discharge diagnosis and not on BP values. While the latter could be the best way to select patients, our ED software doesn’t permit such an extraction. However, we have tried to search for all the disease that could be related to HE or UH. Furthermore, we also evaluated subjects in which discharge diagnosis correspond to symptoms that could be related to HE and UH. Despite our attention to diagnosis definitions the risk of a prevalence underestimation exists for the whole population but also for HE and UH frequency. Thirdly, 383 patients had missing data for the second ED BP values. These patients present less frequently chronic hypertension, and relative therapies, less frequently presents with dyspnea and neurological symptoms and had lower BP values. Finally, they were less frequently treated with some differences in used drugs, particularly intravenous one. A clinical interpretation could be that these subjects present clinical characteristics and lower prevalence of specific symptoms that reduce their probability to present a EH and so physicians are less prone to perform further BP evaluation before discharge. Furthermore we cannot exclude that BP values were measured but not recorded on ED report. This could have determined a possible overestimation of BP reduction regression-to-the-mean and treatment-selection bias.

Same bias could be also determined by the fact that while first ED BP values evaluation was strongly standardized, the further ones were dependent on physicians’ decisions. Although they were not randomly assessed possible further bias can be determined by the absence of a systematic evaluation.

The risk of missing values also applies to biochemical variables. The absence of a systematic laboratory evaluation could have determined an underestimation of some HMOD and particularly of silent acute kidney injury. The same apply also to myocardial injury and heart failure in which a systematic evaluation of troponin and natriuretic peptides will lead to a higher prevalence of the relative disease. However, in these latter cases the probability of underestimation is lower because they are mostly symptomatic.

Another point is that the BP threshold defining HE/UH is not universally accepted [6]. However, the definition adopted in our study (i.e. BP values ≥ 180/120 mmHg) is the one most commonly used in epidemiological research papers [14, 24].

Furthermore, there could be a possible misclassification bias for untreated patients for which therapies could be initiated but not registered in ED or initiated after the specialist evaluation not in the ED setting. Also regarding therapies, we didn’t assess compliance to chronic anti-hypertensive treatment or treatment discontinuation before ED admission, that could be a strong factor related to HE/UH occurrence.

Moreover, total ED stay time were not registered for the present study. Finally, despite the total number of subjects in the whole population, some subgroup presented a low number of subjects (i.e. aortic dissection): this sample size can limit our power to detect important associations or findings. However, this is determined by the heterogeneity of HE and by the rarity of some events.

In conclusion, this study confirms the prevalence of HE/UHs and the variables associated (positively and negatively) to HE. Furthermore, adherence to guidelines, particularly regarding pharmacological approach, could be further improved. Intervention such as electronic decision support systems, automatic health record alert, mandatory ED protocols, telemedical assisted pre-ED intervention, have already been evaluated with positive results [26, 27]. Finally, no association was identified between the management of BP in the ED and in-hospital mortality.

## Supplementary information

Supplementary information
