# Supplementary material for: Prevalence, clinical features and emergency department blood pressure management of hypertensive emergencies and uncontrolled hypertension: a retrospective multi-centre study
Source: Hypertens Res. 2026 Sep 2;49(11):3124–36. doi: 10.1038/s41440-026-02788-1 (PMC13736647; doi:10.1038/s41440-026-02788-1)
Supplement: Supplementary file 1 [file 41440_2026_2788_MOESM1_ESM.docx]

**Supplementary table 1:** Clinical characteristics of the total sample and in patients classified depending on the absence of a second blood pressure measurement.

| **Variable** | **Overall** | **No missing BP values** | **Missing BP values** | **p-value** |
| --- | --- | --- | --- | --- |
| **Number** | 1838 | 1455 | 383 |  |
| **Cardiovascular risk factors** | | | | |
| **Age (years)** | 70.5 ± 14.4 | 70.3 ± 14.7 | 71.2 ± 13.4 | 0.267 |
| **Females** | 985 (53.6%) | 794 (54.5%) | 191 (49.8%) | 0.101 |
| **Smoke** | 203 (11.0%) | 167 (11.4%) | 36 (9.4%) | 0.248 |
| **Diabetes** | 382 (20.8%) | 303 (20.8%) | 79 (20.6%) | 0.932 |
| **Hypertension** | 1,347 (73.3%) | 1,099 (75.5%) | 248 (64.7%) | <0.001 |
| **Chronic antihypertensive treatment** | 1,169 (63.6%) | 953 (65.4%) | 216 (56.3%) | <0.001 |
| **Two or more antihypertensive drugs** | 747 (40.6%) | 602 (41.3%) | 145 (37.8%) | 0.213 |
| **Cardiovascular and general comorbidities** | | | | |
| **Chronic kidney disease** | 205 (11.2%) | 169 (11.6%) | 36 (9.4%) | 0.220 |
| **Peripheral artery disease** | 290 (15.8%) | 236 (16.2%) | 54 (14.1%) | 0.311 |
| **Previous acute coronary syndrome** | 368 (20.0%) | 300 (20.6%) | 68 (17.7%) | 0.213 |
| **Previous heart failure** | 117 (6.4%) | 103 (7.1%) | 14 (3.7%) | 0.015 |
| **Chronic obstructive pulmonary disease** | 110 (6.0%) | 89 (6.1%) | 21 (5.5%) | 0.642 |
| **Asthma** | 32 (1.7%) | 25 (1.7%) | 7 (1.8%) | 0.884 |
| **Previous stroke** | 147 (8.0%) | 108 (7.4%) | 39 (10.1%) | 0.076 |
| **Cancer** | 206 (11.2%) | 166 (11.4%) | 40 (10.4%) | 0.594 |
| **Emergency department symptoms** | | | | |
| **Symptoms at presentation** | 1,625 (88.4%) | 1,285 (88.3%) | 340 (88.7%) | 0.804 |
| **Chest pain** | 328 (17.8%) | 252 (17.3%) | 76 (19.8%) | 0.251 |
| **Dyspnea** | 327 (17.8%) | 273 (18.7%) | 54 (14.1%) | 0.034 |
| **Syncope** | 28 (1.5%) | 20 (1.4%) | 8 (2.1%) | 0.310 |
| **Neurological Deficit** | 420 (22.9%) | 317 (21.7%) | 103 (26.8%) | 0.034 |
| **Palpitation** | 73 (4.0%) | 58 (4.0%) | 15 (3.9%) | 0.950 |
| **Headaches** | 367 (20.0%) | 315 (21.6%) | 52 (13.6%) | <0.001 |
| **Vertigo** | 260 (14.1%) | 225 (15.4%) | 35 (9.1%) | 0.002 |
| **Vomit** | 118 (6.4%) | 98 (6.7%) | 20 (5.2%) | 0.282 |
| **Epistaxis** | 118 (6.4%) | 87 (6.0%) | 31 (8.1%) | 0.133 |
| **Abdominal pain** | 25 (1.4%) | 22 (1.5%) | 3 (0.8%) | 0.273 |
| **Presentation BP values** | | | | |
| **Systolic BP (mmHg)** | 194.5 ± 15.1 | 196.3 ± 15.6 | 187.5 ± 10.6 | <0.001 |
| **Diastolic BP (mmHg)** | 97.0 ± 16.4 | 98.7 ± 16.8 | 90.6 ± 13.0 | <0.001 |
| **Hypertension Emergency** | 693 (37.7%) | 553 (38.0%) | 140 (36.6%) | 0.602 |
| **Pharmacological treatment** | | | | |
| **Pharmacological treatment in ED** | 979 (53%) | 106 (28%) | 873 (60%) | <0.001 |
| **IV nitroglycerin** | 102 (5.5%) | 9 (2.3%) | 93 (6.4%) | 0.002 |
| **Nitroglycerin sublingual** | 45 (2.4%) | 5 (1.3%) | 40 (2.7%) | 0.135 |
| **Metoprolol** | 19 (1.0%) | 3 (0.8%) | 16 (1.1%) | 0.779 |
| **Labetalol** | 47 (2.6%) | 0 (0%) | 47 (3.2%) | <0.001 |
| **Urapidil** | 20 (1.1%) | 2 (0.5%) | 18 (1.2%) | 0.403 |
| **Furosemide** | 273 (15%) | 41 (11%) | 232 (16%) | 0.010 |
| **Clonidine** | 222 (12%) | 15 (3.9%) | 207 (14%) | <0.001 |
| **Bisoprolol** | 17 (0.9%) | 1 (0.3%) | 16 (1.1%) | 0.225 |
| **Atenolol** | 11 (0.6%) | 0 (0%) | 11 (0.8%) | 0.134 |
| **Captopril** | 67 (3.6%) | 1 (0.3%) | 66 (4.5%) | <0.001 |
| **Ramipril** | 21 (1.1%) | 2 (0.5%) | 19 (1.3%) | 0.282 |
| **Enalapril** | 25 (1.4%) | 2 (0.5%) | 23 (1.6%) | 0.138 |
| **Nifedipine short acting** | 150 (8.2%) | 4 (1.0%) | 146 (10%) | <0.001 |
| **Nifedipine long acting** | 12 (0.7%) | 0 (0%) | 12 (0.8%) | 0.083 |
| **Amlodipine** | 151 (8.2%) | 13 (3.4%) | 138 (9.5%) | <0.001 |
| **Doxazosin** | 12 (0.7%) | 0 (0%) | 12 (0.8%) | 0.083 |
| **Benzodiazepines** | 147 (8.0%) | 23 (6.0%) | 124 (8.5%) | 0.113 |

Data are shown as mean and standard deviation or number and frequency for continuous and categorical one, respectively. Welch Two Sample t-test or Fisher's exact test were used for comparison. Abbreviations: BP = Blood Pressure; ED = Emergency Department.

**Supplementary table 2:** Blood pressure data of hospitalized vs not-hospitalized patients, hospitalized HE vs UH and survivor’s vs in-hospital death.

| **BP values in hospitalized vs not hospitalized patients** | | | | | |
| --- | --- | --- | --- | --- | --- |
| **Variable** | **Overall** | | **Not hospitalized** | **Hospitalized** | **p-value** |
| **Number** | 1,838 | | 1,227 | 611 | - |
| **SBP at presentation (mmHg)** | 196.1 ± 16.5 | | 195.9 ± 16.2 | 200.9 ± 20.69 | 0.217 |
| **DBP at presentation (mmHg)** | 96.5 ± 16.5 | | 96.6 ± 16.4 | 95.7 ± 19.7 | 0.811 |
| **SBP decrease (mmHg)** | 40.6 ± 27.0 | | 40.9 ± 26.8 | 33.7 ± 33.1 | 0.391 |
| **DBP decrease (mmHg)** | 17.0 ± 18.0 | | 17.0 ± 17.9 | 13.3 ± 23.2 | 0.513 |
| **Missing for SBP and DBP decrease** | 383 | | 258 | 125 | - |
| **Hypertensive emergencies** | 693 (37.7%) | | 133 (10.8%) | 560 (91.6%) | <0.001 |
| **BP values and hospitalization related variables between HE and UH only in Hospitalized subjects** | | | | | |
| **Variable** | **Overall** | **Hypertensive emergencies** | | **Uncontrolled hypertension** | **p-value** |
| **Number** | 611 | 560 | | 51 | - |
| **Days of hospitalization** | 10.8 ± 8.5 | 10.7 ± 7.4 | | 11.6 ± 16.0 | 0.702 |
| **In-hospital mortality** | 28 (4.6%) | 27 (4.8%) | | 1 (2.0%) | 0.501 |
| **SBP at presentation (mmHg)** | 196.1 ± 16.5 | 196.1 ± 16.3 | | 196.7 ± 18.3 | 0.814 |
| **DBP at presentation (mmHg)** | 96.5 ± 16.5 | 96.7 ± 16.3 | | 95.2 ± 19.1 | 0.600 |
| **SBP decrease (mmHg)** | 40.6 ± 27.0 | 40.6 ± 27.1 | | 40.2 ± 25.7 | 0.924 |
| **DBP decrease (mmHg)** | 16.9 ± 18.1 | 17.0 ± 17.9 | | 16.0 ± 19.1 | 0.762 |
| **Missing for SBP and DBP decrease** | 125 | 110 | | 15 | - |
| **BP values with patients categorized depending on in-hospital death** | | | | | |
| **Variable** | **Overall** | **Survivors** | | **In-hospital death** | **p-value** |
| **Number** | 611 | 583 | | 28 | - |
| **SBP at presentation (mmHg)** | 196.1 ± 16.5 | 195.9 ± 16.2 | | 200.9 ± 20.6 | 0.217 |
| **DBP at presentation (mmHg)** | 96.5 ± 16.5 | 96.6 ± 16.4 | | 95.7 ± 19.7 | 0.811 |
| **SBP decrease (mmHg)** | 40.6 ± 27.0 | 40.9 ± 26.7 | | 33.7 ± 33.1 | 0.391 |
| **DBP decrease (mmHg)** | 16.7 ± 18.1 | 17.1 ± 17.9 | | 13.3 ± 23.2 | 0.513 |
| **Missing for SBP and DBP decrease** | 125 | 114 | | 11 |  |
| **Hypertensive emergencies** | 560 (91.6%) | 533 (91.4%) | | 27 (96.4%) | 0.501 |

Data are shown as mean and standard deviation or number and frequency for continuous and categorical one, respectively.

Welch Two Sample t-test or Fisher's exact test were used for comparison.

Abbreviations: BP = Blood Pressure; SBP = Systolic Blood Pressure; DBP = Diastolic Blood Pressure; HE = Hypertensive Emergencies; UH = Uncontrolled Hypertension.

**Supplementary table 3:** Clinical characteristics of the total sample and of untreated vs treated patients with a diagnosis of haemorrhagic strokes.

| **Variable** | **Overall haemorrhagic strokes** | **Untreated** | **Treated** | **p-value** |
| --- | --- | --- | --- | --- |
| **Number** | 47 | 21 | 26 |  |
| **Cardiovascular risk factors** | | | | |
| **Age (years)** | 71.0 ± 14.0 | 75.6 ± 11.2 | 67.3 ± 15.2 | 0.101 |
| **Females** | 25 (53.2%) | 13 (61.9%) | 12 (46.1%) | 0.381 |
| **Smoke** | 3 (6.4%) | 1 (4.8%) | 2 (7.7%) | 1 |
| **Diabetes** | 8 (17.0%) | 5 (23.8%) | 3 (11.5%) | 0.437 |
| **Hypertension** | 28 (59.6%) | 12 (57.1%) | 16 (61.5%) | 0.775 |
| **Chronic antihypertensive treatment** | 23 (48.9%) | 12 (57.1%) | 11 (42.3%) | 0.385 |
| **Two or more antihypertensive drugs** | 11 (23.4%) | 5 (23.8%) | 6 (23.1%) | 1 |
| **Cardiovascular and general comorbidities** | | | | |
| **Chronic kidney disease** | 2 (4.3%) | 0 (0%) | 2 (7.7%) | 0.495 |
| **Peripheral artery disease** | 7 (14.8%) | 2 (9.5%) | 5 (19.2%) | 0.436 |
| **Previous acute coronary syndrome** | 6 (12.7%) | 1 (4.8%) | 5 (19.2%) | 0.204 |
| **Previous heart failure** | 1 (2.1%) | 0 (0%) | 1 (3.8%) | 1 |
| **Chronic obstructive pulmonary disease** | 3 (6.4%) | 2 (9.5%) | 1 (3.8%) | 0.579 |
| **Asthma** | 0 (0%) | 0 (0%) | 0 (0%) | - |
| **Previous stroke** | 2 (4.3%) | 1 (4.8%) | 1 (3.8%) | 1 |
| **Cancer** | 7 (14.8%) | 5 (23.8%) | 2 (7.7%) | 0.217 |
| **Emergency department symptoms** | | | | |
| **Symptoms at presentation** | 42 (89.3%) | 17 (80.9%) | 25 (96.1%) | 0.158 |
| **BP values** | | | | |
| **Systolic BP (mmHg)** | 211.3 ± 22.5 | 206.5 ± 22.7 | 215.1 ± 22.0 | 0.156 |
| **Diastolic BP (mmHg)** | 103.0 ± 17.9 | 98.4 ± 19.1 | 106.7 ± 16.3 | 0.163 |
| **Number of patients with a second BP measure** | 35 | 13 | 22 |  |
| **SBP decrease (mmHg)** | 50.3 ± 33.4 | 45.7 ± 30.2 | 53.0 ± 35.5 | 1 |
| **DBP decrease (mmHg)** | 23.8 ± 20.8 | 18.3 ± 21.5 | 27.0 ± 20.2 | 0.329 |
| **Time from the two considered BP evaluations (minutes)** | 156.2 ± 223.8 | 72.5 ± 35.9 | 172.2 ± 241.3 | 0.415 |

Data are shown as mean and standard deviation or number and frequency for continuous and categorical one, respectively. Welch Two Sample t-test or Fisher's exact test were used for comparison. Abbreviations: BP = Blood Pressure.

**Supplementary table 4:** Clinical characteristics of the total sample and of untreated vs treated patients with a diagnosis of acute kidney injury. P-values not calculated due to the low number of untreated patients.

| **Variable** | **Overall acute kidney injury** | **Untreated** | **Treated** |
| --- | --- | --- | --- |
| **Number** | 13 | 6 | 7 |
| **Cardiovascular risk factors** | | | |
| **Age (years)** | 76.8 ± 12.3 | 79.8 ± 10.1 | 74.3 ± 14.3 |
| **Females** | 4 (30.7%) | 2 (33.3%) | 2 (28.5%) |
| **Smoke** | 1 (7.7%) | 0 (0%) | 1 (14.2%) |
| **Diabetes** | 4 (30.7%) | 1 (16.6%) | 3 (42.8%) |
| **Hypertension** | 12 (92.3%) | 6 (100%) | 6 (85.7%) |
| **Chronic antihypertensive treatment** | 12 (92.3%) | 6 (100%) | 6 (85.7%) |
| **Two or more antihypertensive drugs** | 7 (53.8%) | 3 (50.0%) | 4 (57%) |
| **Cardiovascular and general comorbidities** | | | |
| **Chronic kidney disease** | 8 (61.5%) | 5 (83.3%) | 3 (42.8%) |
| **Peripheral artery disease** | 3 (23.0%) | 1 (16.%) | 2 (28.5%) |
| **Previous acute coronary syndrome** | 3 (23.0%) | 1 (16.6%) | 2 (28.5%) |
| **Previous heart failure** | 3 (23.0%) | 0 (0%) | 3 (42.8%) |
| **Chronic obstructive pulmonary disease** | 3 (23.0%) | 2 (33.3%) | 1 (14.2%) |
| **Asthma** | 1 (7.7%) | 1 (16.6%) | 0 (0%) |
| **Previous stroke** | 2 (15.4%) | 1 (16.%) | 1 (14.2%) |
| **Cancer** | 2 (15.4%) | 1 (16.%) | 1 (14.2%) |
| **Emergency department symptoms** | | | |
| **Symptoms at presentation** | 11 (84.6%) | 6 (100%) | 5 (71.4%) |
| **BP values** | | | |
| **Systolic BP (mmHg)** | 191.5 ± 17.8 | 185.0 ± 8.4 | 197.1 ± 22.3 |
| **Diastolic BP (mmHg)** | 94.2 ± 19.8 | 86.7 ± 15.4 | 100.7 ± 21.9 |
| **Number of patients with a second BP measure** | 8 | 2 | 6 |
| **SBP decrease (mmHg)** | 37.1 ± 22.7 | 37.5 ± 3.5 | 37.0 ± 26.8 |
| **DBP decrease (mmHg)** | 15.3 ± 10.5 | 25.0 ± 7.1 | 12.0 ± 9.7 |
| **Time from the two considered BP evaluations (minutes)** | 321.7 ± 402.9 | NA | 321.7 ± 402.9 |

Data are shown as mean and standard deviation or number and frequency for continuous and categorical one, respectively. Welch Two Sample t-test or Fisher's exact test were used for comparison. Abbreviations: BP = Blood Pressure.

**Supplementary table 5:** Clinical characteristics of the total sample and of untreated vs treated patients with a diagnosis of acute pulmonary oedema. P-values not calculated due to the low number of untreated patients.

| **Variable** | **Overall acute pulmonary oedema** | **Untreated** | **Treated** |
| --- | --- | --- | --- |
| **Number** | 76 | 2 | 74 |
| **Cardiovascular risk factors** | | | |
| **Age (years)** | 78.3 ± 9.8 | 80.5 ± 7.8 | 78.3 ± 9.9 |
| **Females** | 44 (57.9%) | 1 (50.0%) | 43 (58.1%) |
| **Smoke** | 11 (14.4%) | 0 (0%) | 11 (14.8%) |
| **Diabetes** | 31 (40.7%) | 0 (0%) | 31 (41.8%) |
| **Hypertension** | 62 (81.6%) | 0 (0%) | 62 (83.7%) |
| **Chronic antihypertensive treatment** | 64 (84.2%) | 0 (0%) | 64 (86.4%) |
| **Two or more antihypertensive drugs** | 52 (68.4%) | 0 (0%) | 52 (70.2%) |
| **Cardiovascular and general comorbidities** | | | |
| **Chronic kidney disease** | 24 (31.5%) | 0 (0%) | 24 (32.4%) |
| **Peripheral artery disease** | 19 (25.0%) | 1 (50.0%) | 18 (24.3%) |
| **Previous acute coronary syndrome** | 29 (38.1%) | 0 (0%) | 29 (39.1%) |
| **Previous heart failure** | 23 (30.2%) | 0 (0%) | 23 (31.1%) |
| **Chronic obstructive pulmonary disease** | 17 (22.3%) | 0 (0%) | 17 (22.9%) |
| **Asthma** | 1 (1.3%) | 0 (0%) | 1 (1.4%) |
| **Previous stroke** | 6 (7.9%) | 0 (0%) | 6 (8.1%) |
| **Cancer** | 17 (22%) | 1 (50.0%) | 16 (22%) |
| **Emergency department symptoms** | | | |
| **Symptoms at presentation** | 76 (100%) | 2 (100%) | 74 (100%) |
| **BP values** | | | |
| **Systolic BP (mmHg)** | 197.8 ± 18.2 | 180.0 ± 0.0 | 198.3 ± 18.3 |
| **Diastolic BP (mmHg)** | 103.9 ± 18.0 | 92.5 ± 17.7 | 104.2 ± 18.0 |
| **Number of patients with a second BP measure** | 73 | 2 | 71 |
| **SBP decrease (mmHg)** | 54.3 ± 33.6 | 7.5 ± 17.7 | 55.6 ± 33.0 |
| **DBP decrease (mmHg)** | 28.6 ± 24.7 | 2.5 ± 10.6 | 29.3 ± 24.7 |
| **Time from the two considered BP evaluations (minutes)** | 179.1 ± 215.0 | 1,420.0 ± NA | 160.9 ± 153.6 |

Data are shown as mean and standard deviation or number and frequency for continuous and categorical one, respectively. Welch Two Sample t-test or Fisher's exact test were used for comparison. Abbreviations: BP = Blood Pressure.

**Supplementary table 6:** Clinical characteristics of the total sample and of untreated vs treated patients with a diagnosis of heart failure.

| **Variable** | **Overall heart failure** | **Untreated** | **Treated** | **p-value** |
| --- | --- | --- | --- | --- |
| **Number** | 216 | 26 | 190 | - |
| **Cardiovascular risk factors** | | | | |
| **Age (years)** | 77.3 ± 11.5 | 74.9 ± 11.5 | 77.6 ± 11.4 | 0.155 |
| **Females** | 105 (48.6%) | 10 (38.4%) | 95 (50.0%) | 0.301 |
| **Smoke** | 31 (14.3%) | 3 (11.5%) | 28 (14.7%) | 1 |
| **Diabetes** | 85 (39.3%) | 7 (26.9%) | 78 (41.0%) | 0.202 |
| **Hypertension** | 168 (77.7%) | 18 (69.2%) | 150 (78.9%) | 0.313 |
| **Chronic antihypertensive treatment** | 186 (86.1%) | 22 (84.6%) | 164 (86.3%) | 0.766 |
| **Two or more antihypertensive drugs** | 153 (70.8%) | 20 (76.9%) | 133 (70.0%) | 0.646 |
| **Cardiovascular and general comorbidities** | | | | |
| **Chronic kidney disease** | 86 (39.8%) | 12 (46.1%) | 74 (38.9%) | 0.525 |
| **Peripheral artery disease** | 56 (22.9%) | 8 (30.7%) | 48 (25.3%) | 0.633 |
| **Previous acute coronary syndrome** | 87 (40.2%) | 8 (30.7%) | 79 (41.5%) | 0.394 |
| **Previous heart failure** | 74 (34.2%) | 8 (30.7%) | 66 (34.7%) | 0.827 |
| **Chronic obstructive pulmonary disease** | 43 (19.9%) | 4 (15.3%) | 39 (20.5%) | 0.793 |
| **Asthma** | 6 (2.8%) | 2 (7.7%) | 4 (2.1%) | 0.154 |
| **Previous stroke** | 23 (10.6%) | 2 (7.7%) | 21 (11.1%) | 1 |
| **Cancer** | 44 (20.3%) | 7 (26.9%) | 37 (19.5%) | 0.435 |
| **Emergency department symptoms** | | | | |
| **Symptoms at presentation** | 209 (96.7%) | 24 (92.3%) | 185 (97.3%) | 0.200 |
| **BP values** | | | | |
| **Systolic BP (mmHg)** | 192.7 ± 15.0 | 188.4 ± 9.7 | 193.3 ± 15.5 | 0.146 |
| **Diastolic BP (mmHg)** | 95.1 ± 17.0 | 88.0 ± 13.3 | 96.0 ± 17.2 | 0.022 |
| **Number of patients with a second BP measure** | 178 | 15 | 163 |  |
| **SBP decrease (mmHg)** | 40.6 ± 25.9 | 36.9 ± 31.3 | 41.0 ± 25.4 | 0.713 |
| **DBP decrease (mmHg)** | 18.2 ± 17.6 | 13.7 ± 13.1 | 18.7 ± 18.0 | 0.311 |
| **Time from the two considered BP evaluations (minutes)** | 251.0 ± 223.4 | 1,420.0 ± NA | 243.3 ± 202.8 | 0.087 |

Data are shown as mean and standard deviation or number and frequency for continuous and categorical one, respectively. Welch Two Sample t-test or Fisher's exact test were used for comparison. Abbreviations: BP = Blood Pressure.

**Supplementary table 7:** Clinical characteristics of the total sample and of untreated vs treated patients with a diagnosis of acute coronary syndrome.

| **Variable** | **Overall acute coronary syndrome** | **Untreated** | **Treated** | **p-value** |
| --- | --- | --- | --- | --- |
| **Number** | 117 | 53 | 64 | - |
| **Cardiovascular risk factors** | | | | |
| **Age (years)** | 69.7 ± 11.8 | 69.8 ± 10.3 | 69.5 ± 13.0 | 0.915 |
| **Females** | 45 (38.4%) | 15 (28.3%) | 30 (46.8%) | 0.056 |
| **Smoke** | 32 (27.3%) | 14 (26.4%) | 18 (28.1%) | 1 |
| **Diabetes** | 32 (27.3%) | 15 (28.3%) | 17 (26.5%) | 0.838 |
| **Hypertension** | 95 (81.1%) | 43 (81.1%) | 52 (81.2%) | 1 |
| **Chronic antihypertensive treatment** | 78 (66.6%) | 34 (64.1%) | 44 (68.7%) | 0.694 |
| **Two or more antihypertensive drugs** | 47 (40.1%) | 23 (43.3%) | 24 (37.5%) | 0.572 |
| **Cardiovascular and general comorbidities** | | | | |
| **Chronic kidney disease** | 15 (12.8%) | 3 (5.7%) | 12 (18.7%) | 0.050 |
| **Peripheral artery disease** | 19 (16.2%) | 8 (15.1%) | 11 (17.1%) | 0.806 |
| **Previous acute coronary syndrome** | 49 (41.8%) | 22 (41.4%) | 27 (42.2%) | 1 |
| **Previous heart failure** | 5 (4.3%) | 1 (1.9%) | 4 (6.3%) | 0.375 |
| **Chronic obstructive pulmonary disease** | 8 (6.8%) | 3 (5.7%) | 5 (7.8%) | 0.727 |
| **Asthma** | 3 (2.6%) | 2 (3.8%) | 1 (1.6%) | 0.589 |
| **Previous stroke** | 8 (6.8%) | 6 (11%) | 2 (3.1%) | 0.138 |
| **Cancer** | 12 (10.2%) | 4 (7.5%) | 8 (12.5%) | 0.543 |
| **Emergency department symptoms** | | | | |
| **Symptoms at presentation** | 117 (100%) | 53 (100%) | 64 (100%) | - |
| **BP values** | | | | |
| **Systolic BP (mmHg)** | 192.0 ± 11.6 | 188.6 ± 8.6 | 194.8 ± 13.1 | 0.010 |
| **Diastolic BP (mmHg)** | 96.5 ± 15.3 | 93.8 ± 11.9 | 98.8 ± 17.4 | 0.062 |
| **Number of patients with a second BP measure** | 85 | 33 | 52 |  |
| **SBP decrease (mmHg)** | 38.0 ± 22.8 | 26.6 ± 17.5 | 45.2 ± 22.9 | <0.001 |
| **DBP decrease (mmHg)** | 14.9 ± 15.1 | 10.6 ± 13.7 | 17.5 ± 15.5 | 0.081 |
| **Time from the two considered BP evaluations (minutes)** | 137.3 ± 216.6 | 1,420.0 ± NA | 110.0 ± 106.8 | 0.097 |

Data are shown as mean and standard deviation or number and frequency for continuous and categorical one, respectively. Welch Two Sample t-test or Fisher's exact test were used for comparison. Abbreviations: BP = Blood Pressure.

**Supplementary figure 1:** systolic (panel A) and diastolic (panel B) blood pressure values at T0 and the higher second values registered during ED stay and their relative delta based on treatment (panel C and D).

Abbreviations: SBP = Systolic Blood Pressure; DBP = Diastolic Blood Pressure.
